# Immediate changes in trunk extension and pain following a structured exercise program in high school baseball players with extension-type low back pain: a retrospective observational study

**DOI:** 10.1186/s13102-026-01928-x

**Published:** 2026-08-01

**Authors:** Tetsuya Miyazaki, Tomoyuki Matsui, Yoshikazu Azuma, Kazuya Seo, Sena Yamazaki, Noriyuki Kida, Toru Morihara

**Affiliations:** 1 Marutamachi Rehabilitation Clinic, 12 Nishinokyo Kurumazakacho Nakagyo-ku, Kyoto, 604-8405 Japan; 2 Rakuwakai Kyoto Sports Medical Science Research Institute, 12 Nishinokyo Kurumazakacho Nakagyo-ku, Kyoto, 604-8405 Japan; 3 Doctoral Programs of Biotechnology, Graduate School of Science and Technology, Kyoto Institute of Technology, Hashikami-cho, Matsugasaki, Sakyo-ku, Kyoto, 606-8585 Japan; 4 Rehabilitation Unit, University Hospital, Kyoto Prefectural University of Medicine, 465 Kajii-cho, Kawaramachi-Hirokoji, Kamigyo-ku, Kyoto, 602-8566 Japan; 5 Faculty of Arts and Sciences, Kyoto Institute of Technology, Hashikami-cho, Matsugasaki, Sakyo-ku, Kyoto, 606-8585 Japan; 6 Department of Orthopaedics, Graduate School of Medical Science, Kyoto Prefectural University of Medicine, 465 Kajii-cho, Kawaramachi-Hirokoji, Kamigyo-ku, Kyoto, 602-8566 Japan

**Keywords:** Adolescent athletes, Acute exercise response, Movement-based assessment, Relative pain intensity, Cluster analysis, Sports rehabilitation

## Abstract

**Background:**

Extension-type low back pain (LBP) is common among baseball players, but immediate exercise responses may differ among athletes. Evidence on exercise-specific acute responses in adolescent athletes remains limited. This retrospective observational study examined immediate changes in trunk extension angle and pain following a five-exercise program and explored response patterns.

**Methods:**

Annual medical checkup data from 6,105 male high school baseball players were retrospectively analyzed. Athletes reporting pain-related limitations (score 3 or 4) on a four-point LBP screening item (from no pain to inability to participate) underwent a two-step clinical examination; 54 athletes clinically diagnosed with extension-type LBP were included. The program included Sit-up Keep, Relax the Rectus Abdominis, Abdominal Draw-in Maneuver, Seated Trunk Rotation, and Hip Flexion Exercise. Trunk extension angle and relative pain intensity were assessed before the program and after each exercise. Relative pain intensity was assessed on an 11-point relative scale from 0 to 10, with 0 indicating no pain and 10 representing pre-program pain. Exploratory factor analysis and Ward’s hierarchical cluster analysis used exercise-specific trunk extension changes.

**Results:**

Mean trunk extension angle increased from 60.7 ± 13.9° to 78.4 ± 16.0° after the exercise sequence. Mean relative pain intensity decreased from 10.0 to 2.4 ± 2.8. Exercise-specific analyses showed that the significant immediate responses differed by outcome, with Sit-up Keep showing the largest mean incremental change in trunk extension angle. Exploratory cluster analysis identified three response patterns: an Affected Area Response Pattern, a Non-Affected Area Response Pattern, and a Transversus Abdominis Response Pattern. Because the Transversus Abdominis Response Pattern included only four participants, this pattern should be interpreted cautiously.

**Conclusions:**

A five-exercise program was associated with immediate within-session improvements in trunk extension angle and relative pain intensity. The exploratory response patterns suggest heterogeneity in acute functional responses and may support individualized assessment. Given the retrospective design, no control group, fixed exercise order, and unvalidated relative pain scale, the findings should not be interpreted as treatment efficacy.

**Trial registration:**

Not applicable. This study was a retrospective analysis of data collected during annual medical checkups and was not registered as a clinical trial.

**Supplementary Information:**

The online version contains supplementary material available at https://doi.org/10.1186/s13102-026-01928-x.

## Background

Low back pain (LBP) is a common musculoskeletal disorder in athletes and often results from the cumulative mechanical stress associated with repetitive sports movements. Clinically, LBP can be classified according to the direction of symptom provocation, including extension, flexion, and mixed types [1]. The present study focused on extension-type LBP, which is characterized primarily by pain provoked during lumbar extension. This clinical presentation is conceptually consistent with Sahrmann’s lumbar extension syndrome [2, 3] and O’Sullivan’s active extension pattern [4], both of which describe movement-related subtypes of LBP.

Although sports training induces functional adaptations in muscles, bones, and joints, it may also contribute to muscle hypertonicity, weakness, imbalance, and asymmetry. Previous studies have shown that sport-specific loading can influence spinal alignment and related physical characteristics [5, 6]. In athletes with extension-type LBP, repetitive lumbar extension and rotation may increase mechanical stress on the posterior elements of the lumbar spine, including the facet joints, pars interarticularis, and spinous processes [7, 8]. In clinical practice, however, symptoms are not determined solely by local lumbar pathology; impairments in adjacent regions, such as the thorax and hip joints, may also influence movement patterns and symptom provocation.

Accordingly, management of extension-type LBP may require assessment beyond the painful lumbar region itself. Decreased trunk function in the affected area, together with restricted thoracic or rib mobility and limited hip extension in non-affected areas, may all contribute to extension-related symptoms [1]. These functional problems may involve both flexibility deficits and motor control or strength deficits. Therefore, an exercise approach addressing both affected and non-affected areas may be clinically relevant when examining movement-related symptoms in athletes with extension-type LBP. Previous exercise-based studies for LBP in athletes have mainly examined core-stability or strengthening-based programs [9, 10], whereas few studies have evaluated immediate exercise-specific responses within a clinically defined extension-type LBP subgroup.

However, evidence regarding immediate exercise-specific responses in adolescent athletes with LBP remains particularly limited. Because high school baseball players may present with sport- and age-related functional characteristics that differ from those of adults with chronic LBP, characterizing acute within-session responses may help clarify response heterogeneity before evaluating longer-term treatment efficacy.

Therefore, the aim of this study was to examine immediate changes following a specific structured five-exercise sequence in male high school baseball players with extension-type LBP and to classify response patterns based on functional characteristics. This exercise sequence is referred to as Immediate Body Conditioning (IBC) in the present study. Rather than establishing causal treatment effectiveness, this study was designed to characterize acute responses to a standardized exercise sequence within a clinically defined subgroup of athletes. We hypothesized that athletes with extension-type LBP would show heterogeneous immediate responses to specific exercises, reflecting differences in underlying functional characteristics such as flexibility deficits and motor control or muscle activation impairments.

## Methods

### Study design and participants

This study was a retrospective analysis of data collected during annual medical checkups among male high school baseball players affiliated with the Kyoto Prefectural High School Baseball Federation. The analysis focused on a clinically defined subgroup of athletes with extension-type low back pain (LBP). The study is reported in accordance with the STROBE statement.

Across the three-year study period, 6,105 athletes underwent annual medical checkups. To identify athletes eligible for the present study, a low back pain screening item administered as part of the annual medical checkup was used. This item was developed for annual medical checkups and has not been previously published elsewhere. The item assessed current low back pain and the extent to which pain affected participation in baseball. Responses were recorded on a four-point scale: (1) no pain; (2) pain or discomfort present, but full participation possible; (3) participation possible, but not at full capacity because of pain; and (4) unable to participate because of pain. The English-language version of the low back pain screening item used in the present study is provided as Additional file 1. Based on these responses, 140 athletes who selected option (3) or (4) underwent a two-step clinical examination. In the first step, 6–8 orthopedic surgeons with at least 5 years of clinical experience performed a standardized clinical screening examination. This screening included medical history taking, assessment of pain provocation during active trunk flexion and extension, Kemp’s test, and screening for neurological signs suggestive of lumbar disc herniation or nerve root compression. In the second step, a senior lead physician with more than 20 years of clinical experience reviewed the initial screening findings, re-confirmed the physical examination findings including Kemp’s test, and confirmed the final clinical diagnosis. Athletes were diagnosed with extension-type LBP when pain was provoked primarily during active trunk extension or Kemp’s test and was not exacerbated during flexion.

Inclusion criteria were a diagnosis of “extension-type” LBP, clinically defined as pain provoked primarily during active trunk extension or Kemp’s test (extension-rotation) without exacerbation during flexion. Exclusion criteria included: (1) neurological signs suggestive of lumbar disc herniation or nerve root compression; (2) history of lumbar surgery; and (3) prior medical consultation resulting in a definitive diagnosis such as lumbar spondylolysis or disc herniation. No radiographic or advanced imaging screening, such as plain radiography, computed tomography, or magnetic resonance imaging, was performed as part of the annual medical checkup. Therefore, the diagnosis of extension-type LBP was based on clinical presentation and physical examination findings rather than structural confirmation.

**Fig. 1:**
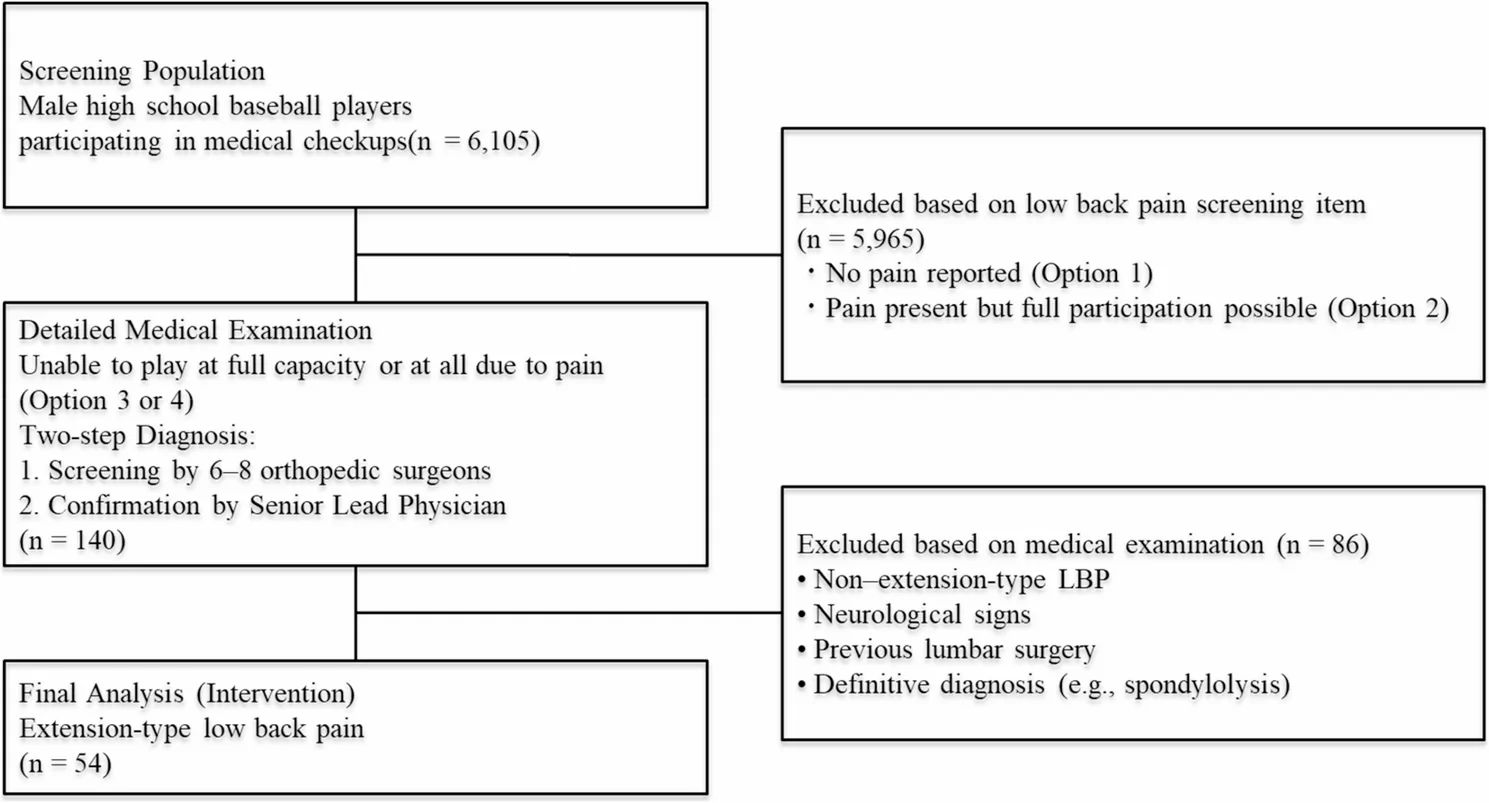
Flowchart of participant selection. During annual medical checkups, 6,105 male high school baseball players were screened using a low back pain screening item. Of these, 140 players who selected option (3) or (4), indicating pain-related limitations in baseball participation, underwent a two-step clinical examination. Finally, 54 players diagnosed with extension-type low back pain met the inclusion criteria and were included in the final analysis.

Ultimately, 54 athletes (mean age: 16.2 ± 0.7 years) met the criteria and were included in the analysis (Fig. 1). For the present dataset, height and weight were not recorded during the annual medical checkups.

### Structured five-exercise program

In the present study, “Immediate Body Conditioning” (IBC) refers to the specific structured five-exercise sequence used for field-based assessment of immediate exercise responses. A precursor whole-body screening and conditioning approach for baseball players with throwing-related disorders has been previously described in Japanese-language reports [11, 12]. However, these reports were not designed to validate the present IBC protocol or to evaluate extension-type LBP. Therefore, the IBC protocol used in the present study should be interpreted as the specific exercise sequence used for within-session assessment rather than as an established or validated clinical program for extension-type LBP.

**Fig. 2:**
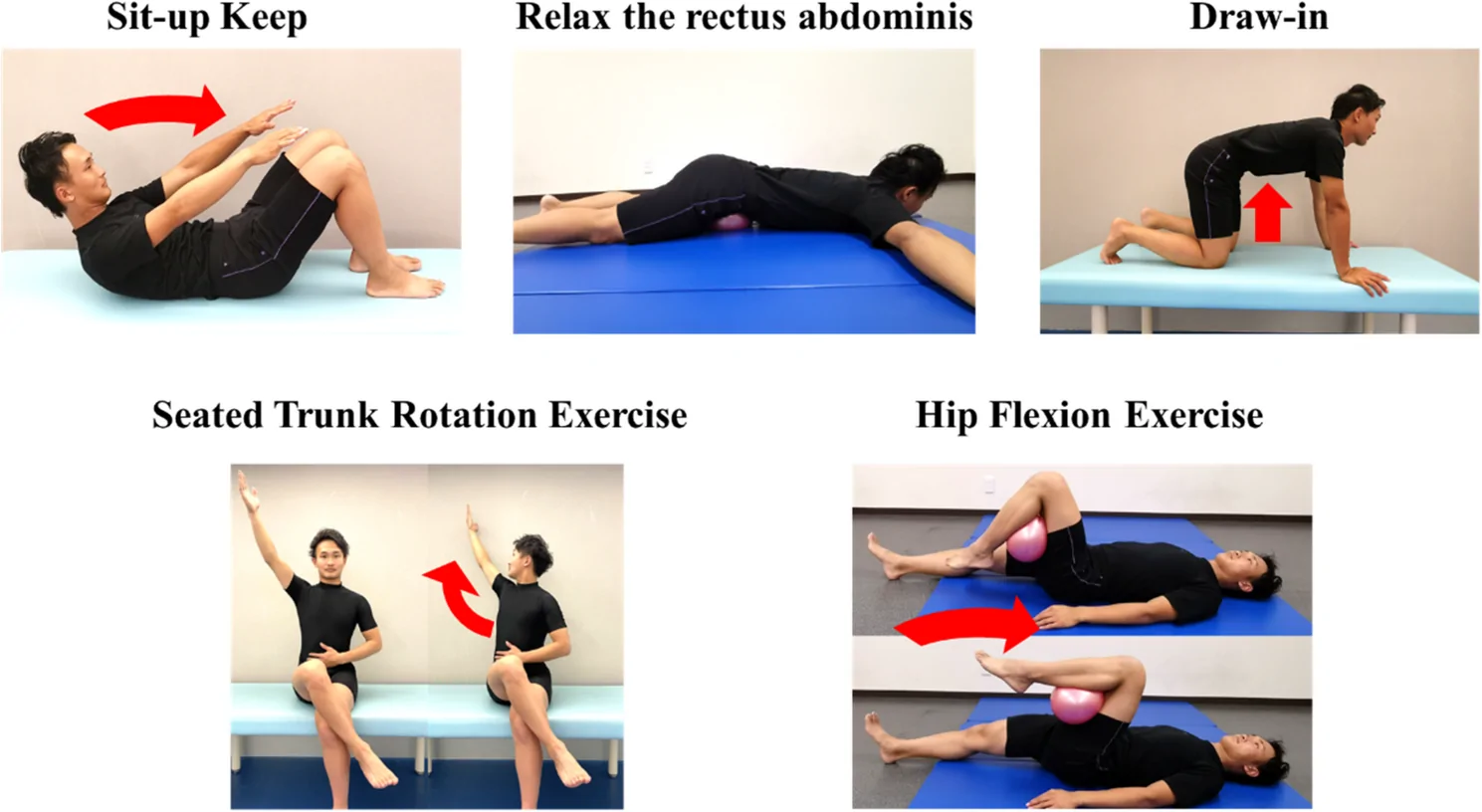
Immediate Body Conditioning (IBC) Program. Sit-up Keep: Lift the upper body to activate the rectus abdominis. Relax the Rectus Abdominis: Place a soft rubber ball under the abdomen and apply sustained pressure to the abdominal muscles to induce relaxation. Draw-in: Flatten the abdomen to activate the transverse abdominis. Seated Trunk Rotation Exercise: Rotate the trunk to activate the serratus posterior inferior. Hip Flexion Exercise: Flex the hip joint to activate the iliopsoas.

The IBC protocol consisted of five specific exercises: Sit-up Keep, Relax the Rectus Abdominis, Abdominal Draw-in Maneuver, Seated Trunk Rotation, and Hip Flexion Exercise (Fig. 2). To ensure reproducibility and consistency, the exercises were instructed by 6–8 physical therapists with at least three years of clinical experience in outpatient sports rehabilitation. A standardized instruction manual was utilized, and consensus training was conducted prior to data collection to unify instruction methods.

The IBC program was performed in a fixed sequence to standardize the within-session assessment and reflect the clinical reasoning used in routine sports rehabilitation. The sequence progressed from exercises intended to reduce excessive superficial trunk muscle tone and pain during extension to exercises targeting trunk muscle activation and mobility of adjacent regions. This fixed order was used for procedural consistency and was not intended to establish the independent efficacy of each exercise or the superiority of this sequence. 

#### Sit-up keep

*Method*: The participant began in a supine position with the hips and knees flexed. The upper body was lifted, and the rectus abdominis was contracted isometrically for 10 s.

*Rationale*: This exercise aims to facilitate rectus abdominis activation and induce reciprocal inhibition of the erector spinae. The rectus abdominis is crucial for motor control during trunk extension [13]; thus, weakness can reduce stability and flexibility in the posterior chain.

#### Relax the rectus abdominis

*Method*: In a prone position, a soft rubber ball approximately 10 cm in diameter was placed under the abdominal wall at three standardized locations around the umbilical region: inferior, central, and superior to the umbilicus. At each location, participants applied pressure using their own body weight while performing deep breathing for 10 s. The same type of ball and standardized placement procedure were used for all participants.

*Rationale*: This aims to relax the rectus abdominis and external oblique muscles. Reduced flexibility in these muscles can impair thoracic and rib mobility required for trunk extension. 

#### Abdominal draw-in maneuver

*Method*: In a quadruped position, participants performed 10 repetitions of pulling the abdomen in while exhaling to activate the transversus abdominis.

*Rationale*: This exercise aims to facilitate transversus abdominis activation [14], which is relevant to lumbar stability during trunk extension [15, 16].

#### Seated trunk rotation

*Method*: In a seated position with one leg crossed and the pelvis stabilized, the ipsilateral arm was raised, and trunk rotations were performed 10 times in both directions.

*Rationale*: This targets the serratus posterior inferior, an accessory breathing muscle [17–19]. Activation promotes lower thoracic cage expansion and thoracic spine mobility.

#### Hip Flexion Exercise

*Method*: In a supine position with hips and knees slightly flexed, active hip flexion was performed 10 times on each side.

*Rationale*: This exercise aims to activate the iliopsoas, given its anatomical and functional relationship with the lumbar spine. It was included to assess the immediate response to hip-focused exercise and to address hip-related motor control that may influence trunk extension movement.

### Outcome measures and procedures

**Fig. 3:**
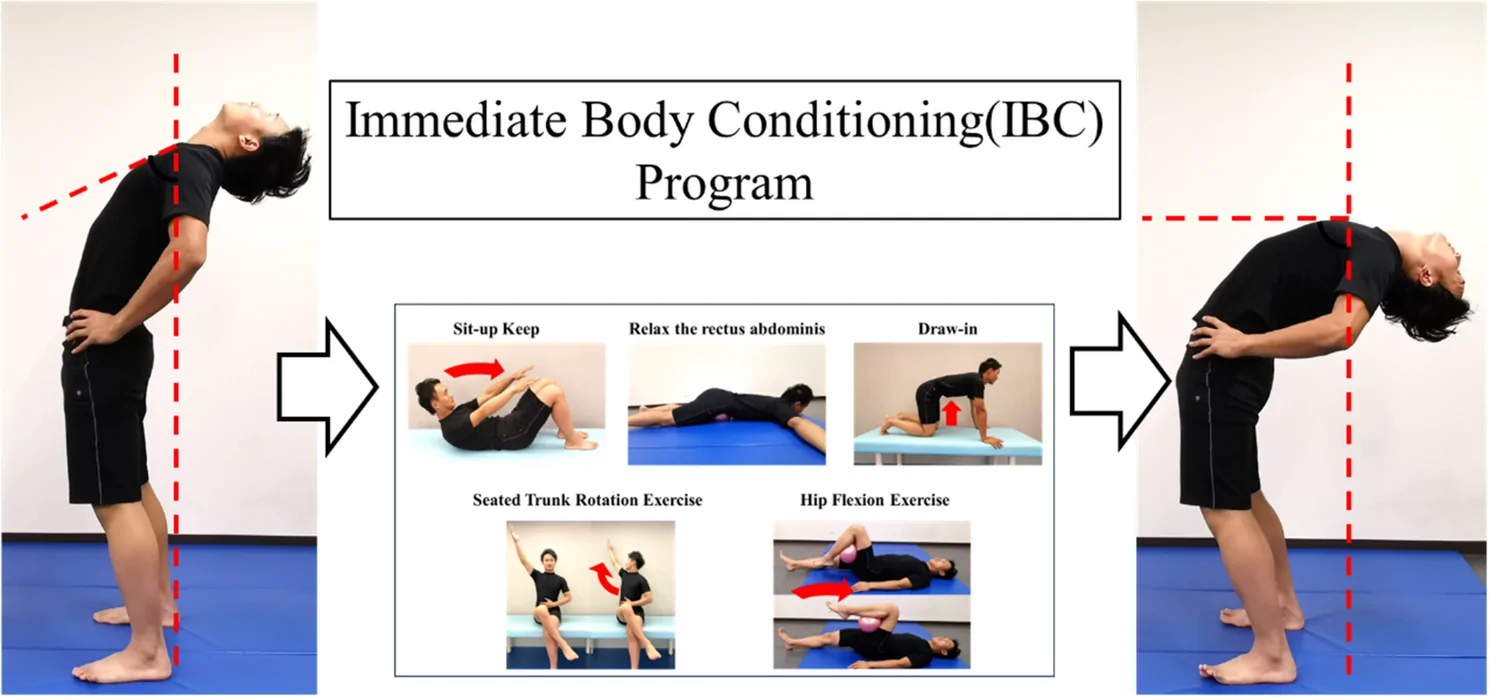
IBC Method. The trunk extension angle is measured with the participant standing with feet shoulder-width apart and hands placed on the hips.

Outcomes were assessed at baseline (pre-IBC) and immediately after each of the five exercises (Fig. 3). Exercise instruction and outcome measurement were performed by different physical therapists. In each assessment sequence, one physical therapist administered the IBC exercise, and another physical therapist measured the trunk extension angle and relative pain intensity immediately after the exercise according to the standardized measurement procedure. Thus, the therapist who instructed the exercise did not perform the immediate post-exercise outcome measurement. However, because the program was conducted in a field-based medical checkup setting using a fixed exercise sequence, the measuring therapist was not blinded to the exercise order or assessment timing. All physical therapists had undergone consensus training prior to the study to ensure consistency.

### Trunk extension angle

The trunk extension angle was measured with the participant standing with feet shoulder-width apart and hands placed on the hips. Participants performed active trunk extension by bending the upper body backward until the onset of pain. A clinometer (Multi-Level; Shinwa Rules Co., Ltd., Japan) was placed on the sternum, and measurements were recorded to the nearest degree. The inter-rater reliability of the trunk extension angle measurement was confirmed in a separate pilot reliability assessment in which two raters measured 10 participants. Reliability was evaluated using the intraclass correlation coefficient (ICC) for single measurements. The ICC for absolute agreement between the measurements obtained by the two raters was 0.956. Based on the same pilot data, the standard error of measurement (SEM) and the minimal detectable change at the 95% confidence level (MDC95) were calculated as indices of measurement error for the trunk extension angle. The SEM was calculated as the square root of the mean square error from the reliability analysis, and the MDC95 was calculated as 1.96 × √2 × SEM. The SEM and MDC95 were 2.24° and 6.20°, respectively.

### Relative pain intensity

Subjective pain during trunk extension was assessed using a custom, non-validated 11-point relative pain scale ranging from 0 to 10. The pre-program pain intensity during trunk extension was defined as 10, and 0 indicated no pain. After each exercise, participants rated their pain relative to the pre-program pain. This scale was used to evaluate immediate within-session changes specific to each exercise and should not be interpreted as a validated numeric rating scale in which 10 represents the worst imaginable pain.

### Measurement protocol

The experimental process followed a repeated sequence: execution of a specific exercise immediately followed by the measurement of the trunk extension angle and relative pain intensity. This cycle was repeated for all five exercises in the fixed order described in the Structured five-exercise program (IBC) section.

### Ethical considerations

This study was conducted in accordance with the Declaration of Helsinki and was approved by the Rakuwakai Research Ethics Review Committee (Protocol Number: Rakugaku-Rin-01-000486). Informed consent was obtained from all participating athletes and their legal guardians.

### Statistical analysis

Statistical analyses were performed using SPSS Statistics version 28 (IBM Corp., Armonk, NY, USA). Data are presented as mean ± standard deviation (SD). Normality was assessed using the Shapiro–Wilk test. There was no missing data for the primary outcome measures.

To evaluate changes across the full exercise sequence, one-way repeated-measures analysis of variance (ANOVA) was used for trunk extension angle and relative pain intensity across six time points (pre-program and after each of the five exercises). When significant main effects were observed, post-hoc multiple comparisons were performed. For exercise-specific pre–post comparisons, paired t-tests were used for trunk extension angle and Wilcoxon signed-rank tests for relative pain intensity. For exercise-specific paired comparisons, Cohen’s dz was calculated as the mean difference divided by the standard deviation of the paired differences to quantify the magnitude of each exercise-specific change. Effect sizes for repeated-measures ANOVA and mixed ANOVA were reported as partial eta squared (η²p). 95% confidence intervals (CI) were calculated where appropriate.

Exploratory factor analysis using the principal factor method was performed on the changes in trunk extension angle to identify latent response patterns. Factor retention was determined based on the scree plot, eigenvalues greater than 1, and interpretability (Additional file 2). Factor scores were calculated using the regression method. Subsequently, Ward’s hierarchical cluster analysis was conducted using these factor scores to classify participants into exploratory response patterns. The number of clusters was determined by examining the dendrogram and the clinical interpretability of candidate cluster solutions. A three-cluster solution was selected because it provided clinically interpretable response patterns; however, because one cluster included only four participants, this cluster solution was considered exploratory. To compare subgroup characteristics, two-way mixed ANOVA was performed (group × exercise). When significant interactions were identified, simple main effects were examined. For exercise-specific pre–post comparisons, the Bonferroni-adjusted significance level was set at *p* < 0.01 to account for multiple comparisons. Because this was a retrospective observational study based on available screening data, no a priori sample size calculation was performed. Two-sided p values < 0.05 were considered statistically significant unless otherwise specified.

## Results

### Overall effect and exercise-specific changes

The IBC program was associated with immediate changes in trunk extension angle and relative pain intensity. The mean trunk extension angle increased from 60.7 ± 13.9° to 78.4 ± 16.0° (+ 17.7°; 29.2% increase). This mean increase exceeded the MDC95 of 6.20° estimated from the separate pilot reliability assessment of the trunk extension angle measurement. The relative pain intensity score decreased from 10.0 to 2.4 ± 2.8 (-7.6 points; 76.0% reduction).

One-way repeated-measures ANOVA revealed significant main effects of time for both trunk extension angle (F(5, 265) = 73.19, *p* < 0.001, η²*p* = 0.58) and relative pain intensity (F(5, 265) = 146.46, *p* < 0.001, η²*p* = 0.73). Exercise-specific post-hoc paired comparisons with Bonferroni correction were then performed separately for trunk extension angle and relative pain intensity.

For trunk extension angle, significant additional increases were observed after “Sit-up Keep,” “Draw-in,” and “Seated Trunk Rotation,” whereas the additional changes after “Relax the Rectus Abdominis” and “Hip Flexion Exercise” did not reach the Bonferroni-adjusted significance threshold. The adjusted p values and effect sizes for trunk extension angle are presented in Table 1. For relative pain intensity, significant additional reductions were observed after “Sit-up Keep,” “Relax the Rectus Abdominis,” “Draw-in,” and “Seated Trunk Rotation,” whereas the additional change after “Hip Flexion Exercise” did not reach the Bonferroni-adjusted significance threshold. The adjusted p values and effect sizes for relative pain intensity are presented in Table 2.

**Table 1:** Exercise-specific post-hoc paired comparisons for trunk extension angle

Comparison	Mean difference	Adjusted *p* value	Cohen’s dz
Sit-up Keep − Baseline	8.37	< 0.001	1.01
Relax the Rectus Abdominis – Sit-up Keep	1.98	0.108	0.38
Abdominal Draw-in Maneuver – Relax the Rectus Abdominis	2.15	0.006	0.51
Seated Trunk Rotation – Abdominal Draw-in Maneuver	2.78	< 0.001	0.84
Hip Flexion Exercise − Seated Trunk Rotation	2.46	0.183	0.35

Legend: Positive mean differences indicate increases in trunk extension angle. Adjusted p values were calculated using Bonferroni correction. Cohen’s dz was calculated as the mean difference divided by the standard deviation of the paired differences

**Table 2:** Exercise-specific post-hoc paired comparisons for relative pain intensity

Comparison	Mean difference	Adjusted *p* value	Cohen’s dz
Sit-up Keep – Baseline	-4.19	< 0.001	1.46
Relax the Rectus Abdominis – Sit-up Keep	-1.06	< 0.001	0.72
Abdominal Draw-in Maneuver – Relax the Rectus Abdominis	-0.83	0.006	0.52
Seated Trunk Rotation – Abdominal Draw-in Maneuver	-0.70	< 0.001	0.76
Hip Flexion Exercise − Seated Trunk Rotation	-0.78	0.17	0.36

Legend: Negative mean differences indicate reductions in relative pain intensity. Adjusted p values were calculated using Bonferroni correction. Cohen’s dz was calculated as the mean difference divided by the standard deviation of the paired differences

**Fig. 4:**
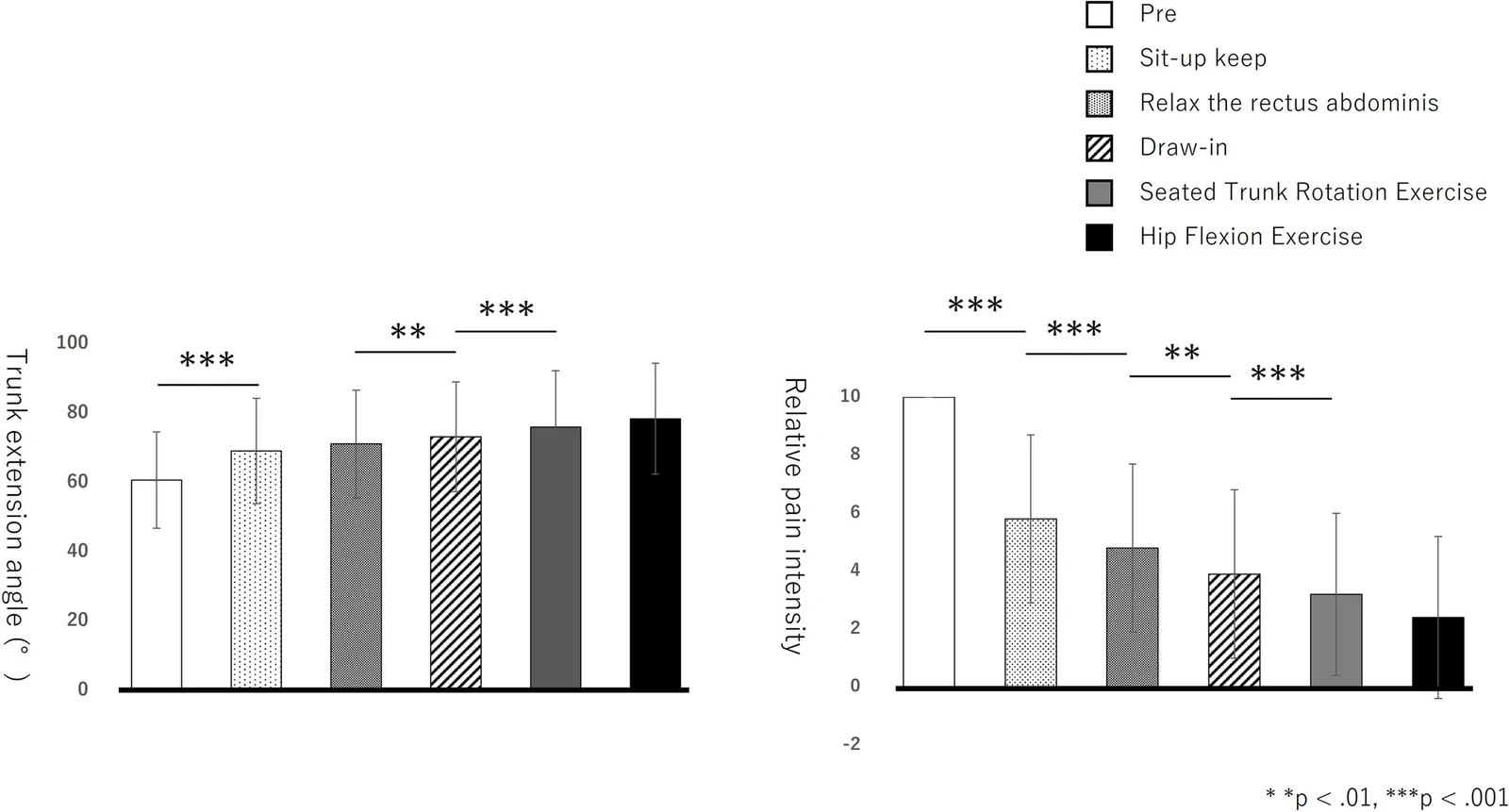
Trunk extension angle (left) and relative pain intensity (right) before the IBC program and after each exercise. Values are presented as mean ± standard deviation. One-way repeated-measures ANOVA with six levels revealed significant main effects of time for both outcomes. For trunk extension angle, post-hoc paired t-tests with Bonferroni correction revealed significant additional increases after “Sit-up Keep,” “Draw-in,” and “Seated Trunk Rotation” (Bonferroni-adjusted p < 0.01), whereas the additional changes after “Relax the Rectus Abdominis” and “Hip Flexion Exercise” did not reach the significance threshold. For relative pain intensity, post-hoc Wilcoxon signed-rank tests with Bonferroni correction revealed significant additional reductions after “Sit-up Keep,” “Relax the Rectus Abdominis,” “Draw-in,” and “Seated Trunk Rotation” (Bonferroni-adjusted p < 0.01), whereas the additional change after “Hip Flexion Exercise” did not reach the significance threshold. Detailed statistics are presented in Tables 1 and 2.

**Fig. 5:**
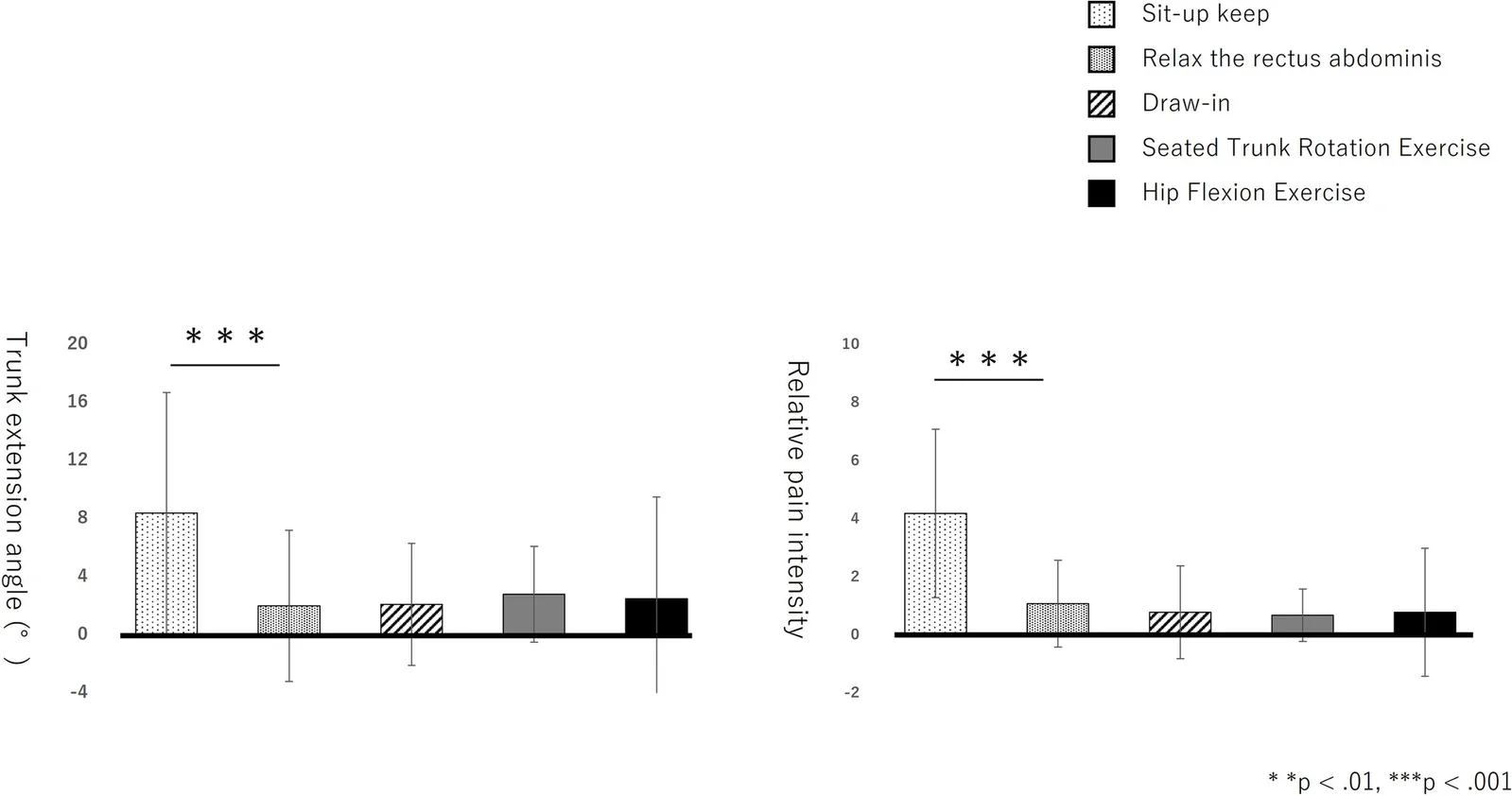
Changes in trunk extension angle (left) and relative pain intensity (right) before the IBC program and after each exercise. Values represent the incremental change after each exercise. For trunk extension angle, significant additional increases were observed after “Sit-up Keep,” “Draw-in,” and “Seated Trunk Rotation” (Bonferroni-adjusted p < 0.01). For relative pain intensity, significant additional reductions were observed after “Sit-up Keep,” “Relax the Rectus Abdominis,” “Draw-in,” and “Seated Trunk Rotation” (Bonferroni-adjusted p < 0.01). Detailed statistics are presented in Tables 1 and 2.

Among the five exercises, “Sit-up Keep” showed the largest mean incremental change in both trunk extension angle and relative pain intensity (Figs. 4 and 5).

### Factor analysis

The exploratory factor analysis using the principal factor method indicated that a three-factor solution was appropriate based on eigenvalues, the slope of the scree plot, and the interpretability of the factors. The eigenvalues were 1.42, 1.18, and 1.03 for the first, second, and third factors, respectively, with a cumulative contribution rate of 72.5%.

Table 3 presents the results of oblique rotation using the Promax method. Factors were defined based on the functional and anatomical characteristics common to the items with high loadings. The first factor was composed of “Draw-in” and “Sit-up Keep.” As these items both involve the active recruitment of the abdominal wall musculature, the factor was defined as the Abdominal Muscle Activation Factor. The second factor consisted of “Relax the Rectus Abdominis,” which targets the inhibition of superficial trunk flexors; thus, it was labeled the Abdominal Muscle Relaxation Factor. The third factor comprised “Seated Trunk Rotation Exercise” and “Hip Flexion Exercise.” Given that these items target the mobility and motor control of the thoracic cage and hip joints—segments adjacent to the lumbar spine—this factor was defined as the Adjacent Joint Control Factor.

**Table 3:** Factor analysis

	Exercises	F1	F2	F3	h2	u2
First Factor	Abdominal Muscle Activation Factor					
	Draw-in	− 0.920	0.329	0.026	0.781	0.219
	Sit-up Keep	0.540	0.270	0.208	0.474	0.526
Second Factor	Abdominal Muscle Relaxation Factor					
	Relax the Rectus Abdominis	0.187	− 0.950	− 0.016	0.833	0.167
Third Factor	Adjacent Joint Control Factor					
	Seated Trunk Rotation Exercise	0.236	0.190	0.830	0.771	0.229
	Hip Flexion Exercise	0.313	0.341	− 0.669	0.766	0.234
	Inter-factor Correlation	F1	0.293	− 0.750		
		F2	-	− 0.023		

Legend: Results of the exploratory factor analysis using oblique rotation with the Promax method for the three extracted factors. The table displays factor loadings (F1, F2, and F3), communalities (h²), uniqueness values, and inter-factor correlations. The three factors were named based on the exercises with high factor loadings: Factor 1, Abdominal Muscle Activation Factor; Factor 2, Abdominal Muscle Relaxation Factor; and Factor 3, Adjacent Joint Control Factor

### Cluster analysis and subgroup characterization

To characterize response heterogeneity, hierarchical cluster analysis using Ward’s method was performed based on the three factor scores derived from exercise-specific changes in trunk extension angle. Based on the dendrogram and clinical interpretability of candidate cluster solutions, a three-cluster solution was selected, and the 54 participants were classified into three exploratory response patterns: Group A (Affected Area Response Pattern, *n* = 31), Group B (Non-Affected Area Response Pattern, *n* = 19), and Group C (Transversus Abdominis Response Pattern, *n* = 4).

**Fig. 6:**
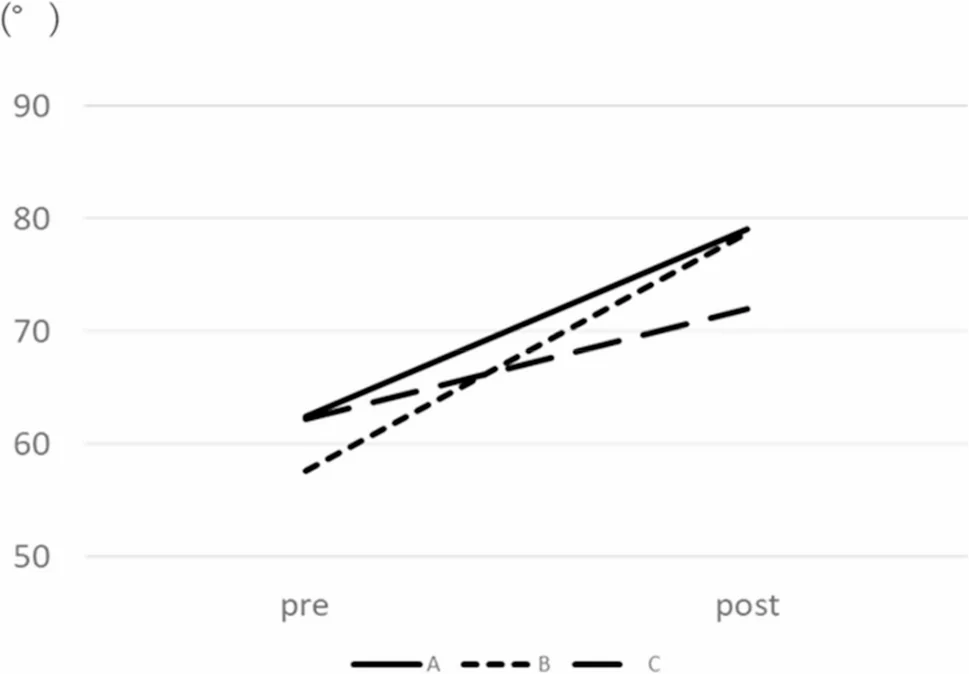
Trunk extension angle before and after the IBC program by cluster group. Values are presented as mean ± standard deviation for each of the three cluster groups (Group A, Affected Area Response Pattern, *n* = 31; Group B, Non-Affected Area Response Pattern, *n* = 19; Group C, Transversus Abdominis Response Pattern, *n* = 4). Two-way mixed ANOVA revealed a significant main effect of time (F(1, 51) = 54.95, *p* < 0.001, η²*p* = 0.519), whereas neither the main effect of cluster group nor the group-by-time interaction was significant.

**Fig. 7:**
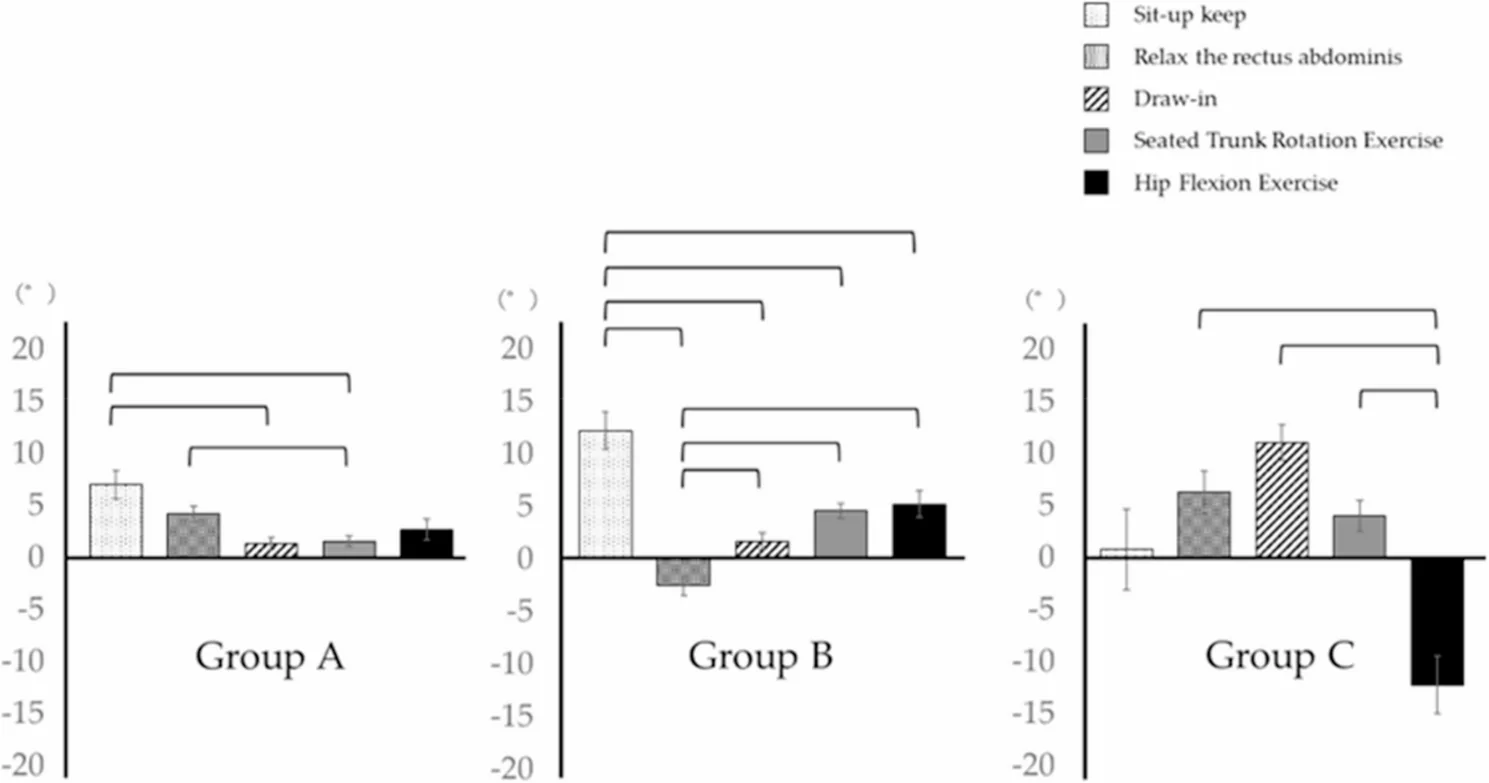
Exercise-specific changes in trunk extension angle by cluster group.Values represent changes in trunk extension angle after each of the five exercises for the three cluster groups. A two-way mixed ANOVA was performed with cluster group as the between-subjects factor, the five exercises as the within-subjects factor, and the change in trunk extension angle as the dependent variable, and revealed a significant group-by-exercise interaction. Simple main effect analyses indicated that Group A showed a significantly larger response than Group B after “Relax the Rectus Abdominis” (Abdominal Muscle Relaxation Factor). Conversely, Group B showed a significantly larger response than Group A after “Seated Trunk Rotation” (Adjacent Joint Control Factor).

For trunk extension angle, a two-way mixed ANOVA (time × cluster group) revealed a significant main effect of time (F(1, 51) = 54.95, *p* < 0.001, η²*p* = 0.519), reflecting the overall increase from pre-program to post-program values. However, neither the main effect of cluster group (F(2, 51) = 0.27, *p* = 0.767, η²*p* = 0.010) nor the group-by-time interaction (F(2, 51) = 2.08, *p* = 0.135, η²*p* = 0.075) was significant, indicating that the three clusters did not differ in trunk extension angle before or after the IBC program (Fig. 6). Similarly, post-program relative pain intensity did not differ significantly among the three clusters (F(2, 51) = 0.33, *p* = 0.720, η²*p* = 0.010; Table 4). In contrast, when the change in trunk extension angle after each exercise was analyzed by two-way mixed ANOVA (exercise × cluster group), the group-by-exercise interaction was highly significant (F(5.41, 137.86) = 12.35, *p* < 0.001, η²*p* = 0.33; Fig. 7), with degrees of freedom adjusted via the Greenhouse–Geisser correction (ε = 0.676).

**Table 4:** Post-program relative pain intensity by cluster group

	Group A (*n* = 31)	Group B (*n* = 19)	Group C (*n* = 4)	F(2, 51)	p	η²p
Relative pain intensity	2.4 ± 2.6	2.3 ± 2.8	3.5 ± 4.4	0.33	0.720	0.010

Legend: Values are presented as mean ± standard deviation. One-way ANOVA showed no significant difference in post-program relative pain intensity among the three groups (F(2, 51) = 0.33, *p* = 0.720, η²*p* = 0.010)

Subsequent simple main effect analyses indicated that the response patterns differed significantly among the clusters at each of the five exercises (all *p* < 0.05; detailed F values are presented in Fig. 7). Post-hoc intra- and inter-cluster pairwise comparisons (Bonferroni-corrected) indicated distinct exercise-specific response patterns among the three groups:

### Group A (affected area response pattern)

Group A showed relatively consistent positive changes across the five exercises. Within this group, Sit-up Keep produced the largest increase in trunk extension angle (7.00° ± 1.39°), which was significantly greater than the changes after Draw-in (MD = 5.71°, *p* = 0.005) and Seated Trunk Rotation (MD = 5.48°, *p* = 0.005).

### Group B (non-affected area response pattern)

Group B showed the largest within-group increase after Sit-up Keep (12.21° ± 1.78°). This group also showed a greater response to Seated Trunk Rotation Exercise (4.58° ± 0.69°) than Group A (1.52° ± 0.54°; MD = 3.06°, *p* = 0.003). In contrast, a negative change was observed after Relax the Rectus Abdominis (-2.53° ± 0.92°), which was significantly lower than the responses to the other exercises within the group (*p* = 0.029) and lower than the responses observed in Groups A and C (*p* < 0.001).

### Group C (transversus abdominis response pattern)

**Fig. 8:**
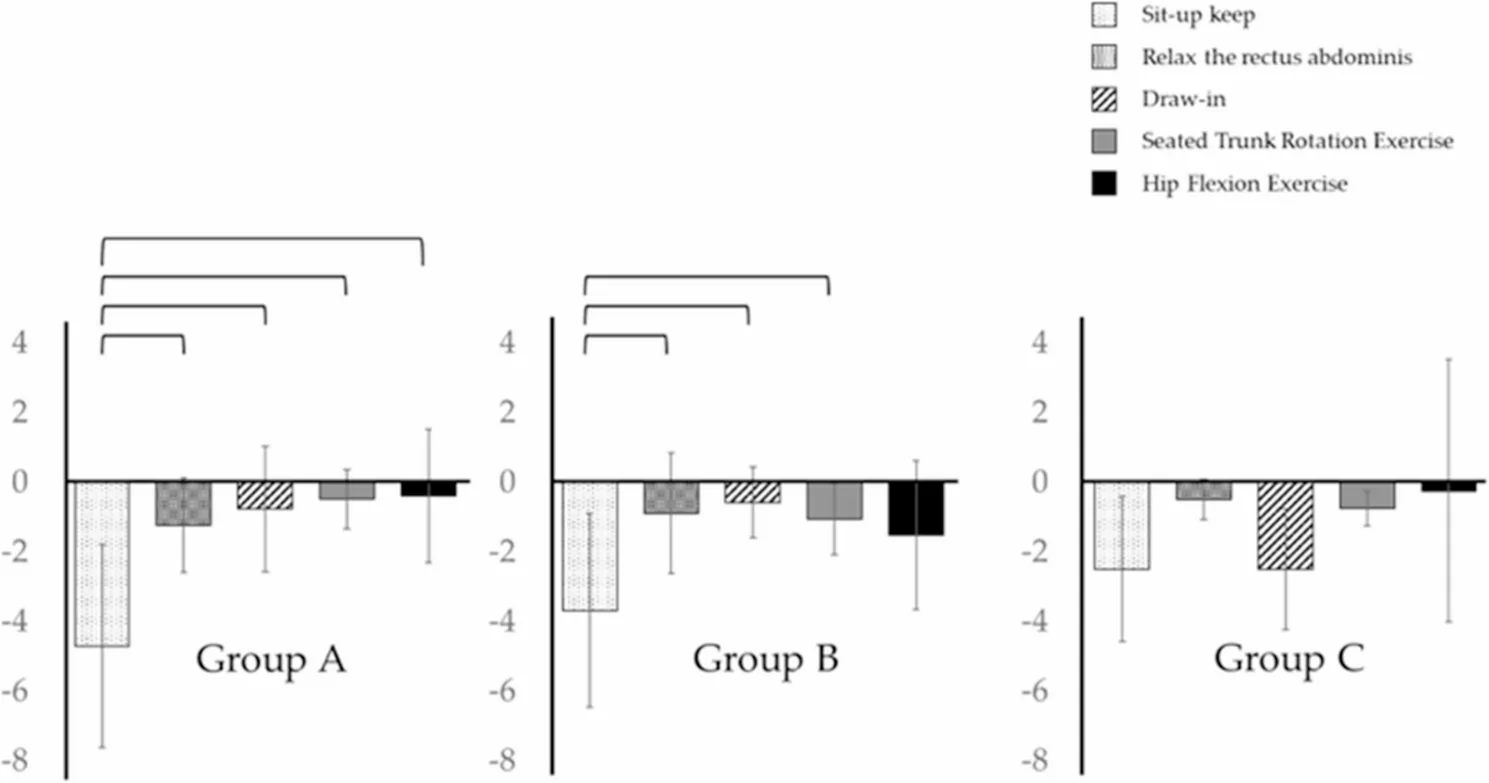
Exercise-specific changes in relative pain intensity by cluster group. Values represent changes in relative pain intensity after each of the five exercises for the three cluster groups. A two-way mixed ANOVA was performed with cluster group as the between-subjects factor, the five exercises as the within-subjects factor, and the change in relative pain intensity as the dependent variable. No significant group-by-exercise interaction was observed.

Group C showed the largest positive change after Draw-in (11.0° ± 1.71°), which was significantly greater than the changes observed in Group A (MD = 9.71°, *p* < 0.001) and Group B (MD = 9.32°, *p* < 0.001). Conversely, a negative change was observed after Hip Flexion Exercise (-12.25° ± 2.77°), which was significantly lower than the responses to the other exercises within the group except Sit-up Keep (*p* < 0.001) and lower than the responses observed in Groups A and B (*p* < 0.001). Because Group C included only four participants, these findings should be interpreted with caution. No significant group-by-exercise interaction was observed for changes in relative pain intensity (Fig. 8).

## Discussion

### Immediate within-session changes following the IBC program

The present study showed that the IBC program was associated with immediate within-session increases in trunk extension angle and reductions in relative pain intensity in male high school baseball players with clinically defined extension-type LBP. Because this was a retrospective observational study without a control group, these findings should be interpreted as immediate associations rather than evidence of treatment efficacy. Nevertheless, the magnitude of the change in trunk extension angle exceeded the MDC95 estimated from the separate pilot reliability assessment, suggesting that the observed change was larger than the estimated measurement error for this outcome.

A practical feature of the IBC program is that it can be completed in approximately 10 min and consists of simple exercises that athletes can perform individually after instruction. Therefore, the present findings may be clinically relevant for field-based sports rehabilitation settings in which rapid within-session assessment is needed. The results of this study do not demonstrate the superiority of any specific single exercise. Rather, the findings suggest that the overall composition and clinical concept of the IBC program may be useful for observing immediate within-session responses in athletes with extension-type LBP.

### Clinical interpretation of exercise-specific responses and response patterns

The exercise-specific findings may be clinically interpreted as reflecting several types of immediate within-session responses. The significant additional increases in trunk extension angle observed after “Sit-up Keep” and “Draw-in” suggest that some athletes may respond to exercises targeting trunk muscle activity and control during extension-provoked movement. This interpretation is consistent with previous reports indicating that abdominal muscle function, including rectus abdominis and transversus abdominis activity, is related to trunk control and LBP [13, 20–22]. However, these responses should be interpreted as immediate within-session changes rather than as evidence of a uniform strengthening effect.

Although pain reduction during trunk extension may be partly related to increased trunk extension range of motion, the exercise-specific findings suggest that this relationship was not uniform. “Relax the Rectus Abdominis” was associated with a significant additional reduction in relative pain intensity but was not associated with a significant additional increase in trunk extension angle. Therefore, its immediate pain-related response may not have been fully explained by a measurable change in range of motion alone, and its independent contribution to trunk extension angle should be interpreted cautiously.

Beyond the local trunk musculature, the significant additional increase in trunk extension angle and significant additional reduction in relative pain intensity observed after “Seated Trunk Rotation” suggest that adjacent regions, particularly the thorax and lower rib cage, may influence extension-provoked symptoms in some athletes. This interpretation is supported by the anatomical relationship between the serratus posterior inferior, lower ribs, and thoracolumbar region [17–19, 23]. In contrast, “Hip Flexion Exercise” was not associated with significant additional changes in either outcome. Although the iliopsoas has anatomical and functional relationships with the lumbar spine [24, 25], the present findings do not support a clear exercise-specific contribution of hip-focused exercise to immediate changes in trunk extension angle or relative pain intensity within this fixed sequence.

The exploratory factor and cluster analyses further suggested that athletes did not respond uniformly to the same exercise sequence. These patterns should not be interpreted as definitive clinical subgroups or as evidence that a specific exercise is superior for a specific subgroup. Rather, they suggest that athletes with clinically defined extension-type LBP may have heterogeneous acute response patterns, and that within-session assessment may help clinicians identify which type of exercise is associated with an immediate response for each athlete. This interpretation is consistent with the broader clinical concept that exercise strategies for LBP may need to account for individual differences in trunk muscle function and motor control [26–28]. However, because the cluster analysis was exploratory and one cluster included only four participants, these findings require prospective validation.

### Generalizability, limitations, and future directions

#### There are several limitations to this study

First, the study employed a retrospective observational design without a control group. Therefore, we cannot completely rule out the possibility that the observed changes were influenced by expectancy effects, repeated testing, familiarization with the movement, or natural fluctuation, nor can we establish a causal relationship.

Second, the exercises were performed in a fixed order for all participants. Although this sequence was intentionally chosen to ensure procedural consistency and to reflect the clinical reasoning used in routine sports rehabilitation, an order effect may have influenced the results. In particular, by the time participants reached the fifth exercise, “Hip Flexion Exercise,” they had already completed four exercises and repeated trunk extension measurements several times. Therefore, measurement burden and within-session fatigue may have contributed to the diminished response to the fifth exercise, independent of the inherent efficacy of the exercise itself. In addition, learning, familiarization, or carryover effects from earlier exercises cannot be ignored. Future studies should consider randomized crossover designs or counterbalanced exercise sequences to isolate the effects of individual exercises.

Third, although SEM and MDC95 were calculated from a separate pilot reliability assessment of the trunk extension angle measurement, intra-rater reliability was not assessed. The repeated within-session measurements in the present study were designed to evaluate immediate exercise responses rather than measurement reliability; therefore, they were not suitable for estimating ICCs because true changes in trunk extension angle could occur after each exercise. In addition, therapist-related variability in exercise instruction and outcome measurement could not be fully evaluated. Although exercise instruction and outcome measurement were performed by different physical therapists, the measuring therapist was not blinded to the exercise order or assessment timing because of the field-based fixed-sequence design.

Fourth, relative pain intensity was assessed using a custom, non-validated 11-point relative scale in which pre-program pain during trunk extension was fixed at 10. Although this approach allowed rapid assessment of immediate within-session changes during large-scale medical checkups, it differs from validated conventional pain instruments. Because all participants were anchored at 10 at baseline regardless of their absolute initial pain intensity, the results may have been influenced by regression to the mean and ceiling effects, particularly in athletes with lower initial pain. In addition, this scale could not capture pain worsening beyond the pre-program level. Therefore, the observed reduction in relative pain intensity should be interpreted cautiously. Future prospective studies should use validated pain measures and record absolute baseline pain intensity to evaluate clinically meaningful changes.

Fifth, regarding selection bias, participants were identified through a comprehensive screening of all high school baseball players in Kyoto Prefecture, rather than being recruited through convenience sampling. However, the final analysis was limited to those who explicitly reported pain in the initial survey and were selected for detailed examination based on specific criteria. While this selection process was based on objective symptom criteria rather than researcher preference, potential response bias in the initial survey, such as athletes with mild pain not reporting their symptoms, cannot be completely excluded.

Sixth, the diagnosis of extension-type LBP was based on clinical presentation and physical examination findings, without radiographic or advanced imaging confirmation. This is an important limitation because pain provoked during trunk extension or Kemp’s test may also be consistent with lumbar spondylolysis, pars interarticularis stress fractures, or other structural pathologies that are common in adolescent athletes. Therefore, undiagnosed structural lesions may have been present in some participants and could have influenced trunk extension range of motion, pain responses, and the identified response patterns. Accordingly, the present findings should be interpreted as responses in athletes with clinically defined extension-provoked LBP rather than as responses specific to a particular structural diagnosis. Future studies should include imaging-based evaluation when feasible to clarify the underlying pathology and examine whether response patterns differ according to structural diagnosis.

Seventh, height and weight were not recorded during the annual medical checkups. Therefore, body mass index and other anthropometric characteristics could not be calculated, which limited our ability to fully characterize the study sample. In addition, because body size and body composition may influence trunk extension range of motion and exercise-specific responses, we could not examine whether these factors affected baseline trunk extension angle, pain responses, or the identified response patterns. Future prospective studies should include height, weight, and body composition-related measures to better characterize participants and evaluate their potential influence on exercise responses.

Eighth, the generalizability of the findings is limited to male high school baseball players. It remains unclear whether similar responses would be observed in female athletes, other age groups, or athletes involved in different sports.

Despite these limitations, this study provides hypothesis-generating insights into immediate exercise-specific responses in athletes with clinically defined extension-type LBP. Future prospective studies with randomized exercise sequences, validated pain measures, imaging-based diagnosis, and long-term follow-up are needed to determine whether these immediate responses translate into sustained pain relief or functional improvement.

## Conclusions

The IBC program was associated with immediate within-session increases in trunk extension angle and reductions in relative pain intensity in male high school baseball players with clinically defined extension-type LBP. Exercise-specific and exploratory cluster findings suggested that athletes may show heterogeneous acute response patterns to the same fixed exercise sequence. These findings may help characterize acute functional responses and support individualized assessment when selecting exercise strategies for athletes with extension-type LBP. However, because this was a retrospective observational study without a control group, the findings should be interpreted as hypothesis-generating rather than as evidence of treatment efficacy or definitive clinical subgroups. Future prospective controlled studies with randomized or counterbalanced exercise sequences, validated pain measures, imaging-based diagnosis, and long-term follow-up are needed to determine whether these immediate responses translate into sustained pain relief or functional improvement.

## Supplementary Information

Additional file 1. English-language version of the low back pain screening item used in the present study. This file provides the English-language version of the low back pain screening item used to identify athletes eligible for the present study. The item assessed current low back pain and its impact on participation in baseball using a four-point response scale.

Additional file 2. Scree plot of eigenvalues obtained from factor analysis. The scree plot displays the eigenvalues for each factor. An inflection point was observed after the third factor, and the eigenvalues of the first three factors exceeded 1.0, supporting the retention of a three-factor solution.

## Data Availability

The datasets generated and/or analyzed during the current study are not publicly available due to the inclusion of personal medical information from adolescent athletes, but are available from the corresponding author on reasonable request and with approval from the relevant institutional ethics committee.

## Abbreviations

ANOVA: Analysis of variance
CI: Confidence interval
IBC: Immediate Body Conditioning
ICC: Intraclass correlation coefficient
LBP: Low back pain
MD: Mean difference
SD: Standard deviation
